# Synthesis of Cu-Doped TiO_2_ on Wood Substrate with Highly Efficient Photocatalytic Performance and Outstanding Recyclability for Formaldehyde Degradation

**DOI:** 10.3390/molecules28030972

**Published:** 2023-01-18

**Authors:** Zhiqiang Lv, Yi Ma, Shanshan Jia, Yan Qing, Lei Li, Yangyang Chen, Yiqiang Wu

**Affiliations:** College of Materials Science and Engineering, Central South University of Forestry and Technology, Changsha 410004, China

**Keywords:** wood composite, Cu-doped TiO_2_, recyclability, formaldehyde degradation, photocatalysis

## Abstract

Photocatalytic oxidation is considered one of the most effective ways to remove formaldehyde from indoor air. However, the use of powder photocatalysts is limited by their low adsorption capacity and strong aggregation tendency. Hence, there is a need for a composite material with good cycling stability and high degradation efficiency. In the present study, a unique wood-based composite is produced by arranging Cu–TiO_2_ nanoparticles on porous structured wood. The porous structure of wood can adsorb formaldehyde, and the abundant functional groups on the surface can act as a reaction platform for anchoring the Cu–TiO_2_ nanoparticles. Cu doping facilitates electron interaction between TiO_2_ and Cu, promotes the transfer of charge carriers, lowers the electron–hole recombination rate, and improves the photocatalytic degradation efficiency of formaldehyde. The photocatalytic efficiency of the wood-based composites was highest (85.59%) when the n(Cu)/n(Ti) ratio was 7%. After nine cycles, the wood composites still had a high degradation rate, indicating good recyclability. Overall, this wood composite is an eco-friendly and promising material for indoor air filtration.

## 1. Introduction

Formaldehyde is a common indoor air pollutant that has been widely used as a component of coatings or adhesives for construction materials and furniture [1,2]. On the other hand, formaldehyde is extremely toxic, can harm the human respiratory and endocrine systems, and can severely limit and affect the safety of the human living environment [3]. Therefore, eliminating formaldehyde pollution from indoor air is an urgent task.

Various techniques have been researched as ways to eliminate formaldehyde from indoor air, such as physical adsorption, plasma oxidation, and photocatalytic oxidation [4,5,6]. Among these treatment technologies, photocatalytic oxidation is considered one of the most promising technologies for the elimination of formaldehyde from indoor air because it is clean, safe, and can decompose formaldehyde to carbon dioxide and water under sunlight [7,8,9]. As a common photocatalyst, titanium dioxide (TiO_2_) has been researched extensively as one of the most promising options for eliminating indoor formaldehyde from indoor air, owing to its strong oxidizing ability, superior photocorrosion resistance, low cost, non-toxicity, and good stability [10,11,12,13]. Nevertheless, its extensive use has been restricted by its low photocatalytic activity, weak adsorption ability, and high aggregation tendency [14,15,16].

One strategy to overcome these constraints is to load TiO_2_ onto porous materials with large surface areas. This structure can efficiently spread the photoactive phase, increase the separation of photogenerated supports, and adsorb the target pollutants near the photocatalytic reaction site [17,18,19,20] (Table S1). Many porous materials, such as carbon materials, sepiolite, and diatomite, have been evaluated as carriers of TiO_2_. However, they contain secondary pollutants or require high-cost precursors [21]. Therefore, finding cost-effective, efficient, and sustainable alternative materials is required. Wood is a type of abundant, renewable, and degradable natural green biomass composite material that can provide a low-cost, long-term carrier for photocatalysts [22]. In addition, wood is a porous, stratified, and well-organized material with abundant voids, which facilitate the absorption of reactants and the diffusion of products [23,24]. Furthermore, the wood surface contains many reactive groups, such as hydroxyl groups, which provide a reaction platform for the anchoring of active substances [25,26]. These advantages allow wood to meet the above-mentioned requirements associated with alternative materials. For instance, Yuan et al. impregnated poplar wood and calcined it at a high temperature to obtain TiO_2_ by using wood as a template. After 280 min of ultra-violet (UV) light irradiation, the breakdown rate of formaldehyde reached only 15.17%. However, its degradation rate was not high, which may have been caused by the poor photocatalytic performance of TiO_2_. Gao et al. [27] successfully coupled Ag/TiO_2_ nanoparticles with wood using a hydrothermal and impregnation approach, which endowed the wood with the ability to achieve the photocatalytic degradation of formaldehyde. In this process, the photocatalytic performance of TiO_2_ is improved by doping TiO_2_ with Ag. However, the recyclability of this wood composite was poor, with the degradation rate decreasing from 94.9% to 2.2% after the fifth cycle. Thus, although some progress has been achieved in the preparation of wood-based composites with degraded formaldehyde, it is important to improve wood-based composites to further improve the recyclability and photocatalytic degradation efficiency of formaldehyde to meet the application objectives.

This work proposes a method to produce Cu–TiO_2_/wood composites that can break down and eliminate formaldehyde under UV-visible (Vis) light. In the composite structure, the wood, as a carrier, could restrict the agglomeration of Cu–TiO_2_ and convey the CO_2_ and H_2_O generated through its vast number of natural gaps. In addition, the abundant functional groups on the wood’s surface provide a platform for anchoring the Cu–TiO_2_ nanoparticles. Benefiting from the synergistic effect, among the wood and Cu-TiO_2_, the wood composite material exhibited good catalytic activity and recyclability, and the synergetic mechanism was discussed. The maximum degradation rate was 85.59% when the Cu/Ti molar ratio was 7%. Additionally, after nine cycles, the wood composite still exhibited a significant formaldehyde breakdown rate, and its reusability was improved greatly. This green and eco-friendly wood composite material allowed the photocatalytic degradation of formaldehyde, which improved the indoor air quality. In addition, this composite material has potential applications in the photocatalytic treatment of indoor formaldehyde gas.

## 2. Results and Discussion

### 2.1. Structural and Morphological Characterization

**Figure 1:**
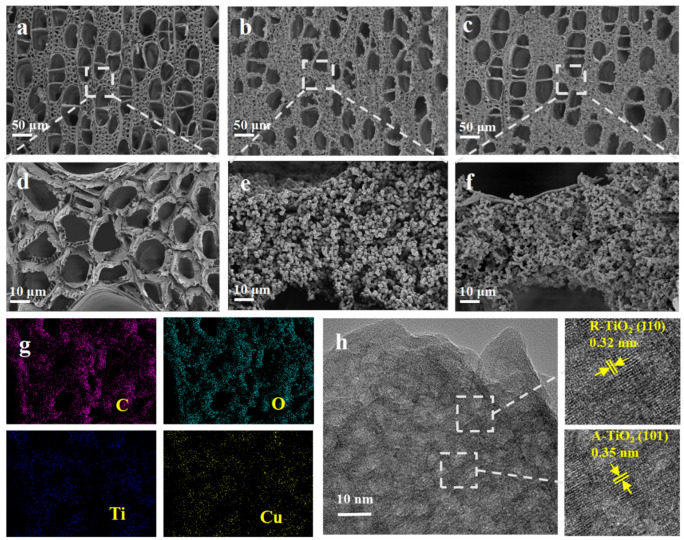
SEM image of the cross-sectional surface of (**a**,**d**) wood, (**b**,**e**) TW, and (**c**,**f**) TWCu7. (**g**) An elemental mapping of TWCu7. (**h**) HRTEM image of the Cu-doped TiO_2_ nanoparticles.

Scanning electron microscopy (SEM) and energy-dispersive spectroscopy (EDS) were used to examine the morphology and microstructures of the Cu–TiO_2_/wood composites. Figure 1a,d shows a cross-sectional view of the microstructure of natural wood. Holes of various sizes were observed in the wood cross section. Large holes 25–100 μm in diameter were formed from the vessels, and other small holes 5–15 μm in diameter were formed by wood fibers or tracheids in the cross section. Figure 1b,c presents SEM images of the TiO_2_/wood (TW) and Cu–TiO_2_/wood (TWCu7). The enormous hole produced by the container can still be seen, but some small holes are hidden because the surface was covered with growing material. Figure 1e,f shows the corresponding high-magnification SEM images of Figure 1b,c, respectively. The altered wood surface was covered with a large number of spherical nanoparticles 500 nm~1 μm in diameter. These spherical nanoparticles produced pores with smaller pore diameters. These pores increased their specific surface area and active sites for interacting with formaldehyde, which can potentially improve the degradation efficiency of formaldehyde. A comparison of the morphologies of TW (Figure 1e) and TWCu7 (Figure 1f) showed that Cu doping did not change the morphologies of the nanoparticles. As shown in the element mapping in Figure 1g, C, O, Ti, and Cu were dispersed over the cross section of the wood. The distributions of Cu and Ti were comparable, suggesting that the TiO_2_ nanoparticles were doped with Cu. Figure 1h presents a high-resolution transmission electron microscopy image of the Cu-doped TiO_2_ nanoparticles. The lattice fringe with a spacing of 0.35 nm was compatible with the interplanar spacing of the anatase TiO_2_ (101), and the lattice fringe with a spacing of 0.32 nm was consistent with the interplanar spacing of rutile TiO_2_ (110). The above data confirm that the Cu–TiO_2_ nanoparticles were anchored successfully to the wood surface.

EDS was used to determine the elemental composition of the sample surface. Figure S1a presents the EDS spectrum corresponding to the original wood. Only the C and O elements that came from wood were detected. Note that the Pt element originated from the coating layer used for SEM observations. In the equivalent EDS of the sample, only C, N, O, F, and Ti were discovered in TW (Figure S1b), whereas Cu was detected in TWCu7 (Figure S1c). The N and F may have been obtained from the (NH_4_)_2_TiF_6_ precursor. The spherical nanoparticles loaded on the wood surface were TiO_2_ because no other elements were detected. EDS further confirmed the presence of Cu-doped TiO_2_ nanoparticles.

**Figure 2:**
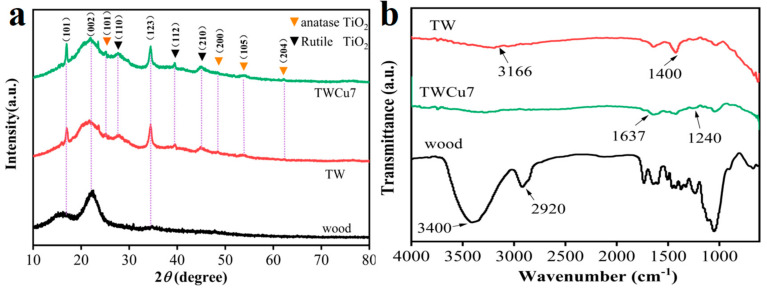
(**a**) XRD patterns of wood, TW, and TWCu7 samples. (**b**) FTIR spectra of wood, TW, and TWCu7 samples.

X-ray diffraction (XRD) was conducted to analyze the chemical composition and crystalline phase of the samples. Figure 2a presents the XRD spectra of the original wood, TW, and TWCu7 samples. All samples showed the (101), (002), and (123) planes at approximately 16.0°, 22.5°, and 34.22°, respectively, which corresponded to the crystal structure of cellulose in wood [28,29]. Seven additional crystal peaks were observed for the TW and TWCu7 samples. The (101), (200), (105), and (204) crystal planes were attributed to anatase TiO_2_ (JCPDS No. 21-1272) [30], and the (110), (112), and (210) planes were assigned to rutile TiO_2_ (JCPDS No. 21-1276) [31], which indicated the presence of anatase and rutile TiO_2_ structures in the samples. However, there was no rutile-type diffraction peak at 2*θ* = 54.32° observed in the figure, indicating that the rutile-type TiO_2_ may be difficult to identify because of its small amount. In addition, the XRD patterns of TW and TWCu7 were similar, with no new diffraction peaks, indicating that Cu doping had a negligible influence on the crystallization properties of TiO_2_ [32].

Fourier transform infrared (FTIR) spectroscopy was performed to examine the functional groups on the sample surfaces, as shown in Figure 2b. In Figure 2b, the absorbance at 3200–3400 cm^−1^ was assigned to the stretching vibration of the hydroxyl group, whereas the vibrational peaks of TW and TWCu7 shifted to lower wavenumbers [33], indicating that the TiO_2_ nanoparticles grown on the wood surface had a strong interaction with the hydroxyl groups on the wood surface through hydrogen bonding. This interaction immobilized the nanoparticle clusters on the wood surface. The peak at 1637 cm^−1^ was assigned to the H–O–H bending vibration of water adsorbed on the sample surface. The hydroxyl groups and water molecules on the sample surface could combine with the photogenerated holes to produce active hydroxyl radicals, boosting the photocatalytic activity. The absorption peak at 1240 cm^−1^ was ascribed to the deformation vibration of the C-H bond and the Ti-O-C stretching vibration. The absorption peak at 2920 cm^−1^ was attributed to the stretching vibration of the –CH_3_ group. The major bands of the TW and TWCu7 samples at 610–800 cm^−1^ were linked to the Ti-O stretching vibration. In addition, the peaks at 1400 and 3166 cm^−1^ were assigned to the bending vibration of the N–H bonds in NH_4_^+^ [34]. This is attributed primarily to the production method of nano-TiO_2_, as illustrated in the formula below:

(1) $$2{\left({\mathrm{NH}}_{2}\right)}_{2}\mathrm{CO}\;+5H_{2}O\;\to 4{\mathrm{NH}}_{4}^{+}+{\;\mathrm{OH}}^{-}+{\;\mathrm{HCO}}_{3}^{-}+{\;\mathrm{CO}}_{3}^{2-}\;$$

(2) $${\left({\mathrm{NH}}_{4}\right)}_{2}{\mathrm{TiF}}_{6}+4{\mathrm{NH}}_{4}^{+}+4{\mathrm{OH}}^{-}\;\to {\;\mathrm{TiO}}_{2}+6{\mathrm{NH}}_{4}F\;+2H_{2}O$$

The absorption peak of NH_4_^+^ also proved that TiO_2_ nanoparticles had formed on the surface of the wood substrate. Compared with TW, there were no clear new distinctive peaks in the infrared spectrum of TWCu7, and the diameters of the absorption peaks in the contrasting regions of the two had changed. This is because after Cu doping, the force between some groups changes, resulting in a change in the width of the absorption peak after doping. Overall, FTIR spectroscopy confirmed that the surface-generated TiO_2_ nanoparticles could attach strongly to wood through hydrogen bonding, resulting in Cu doping.

**Figure 3:**
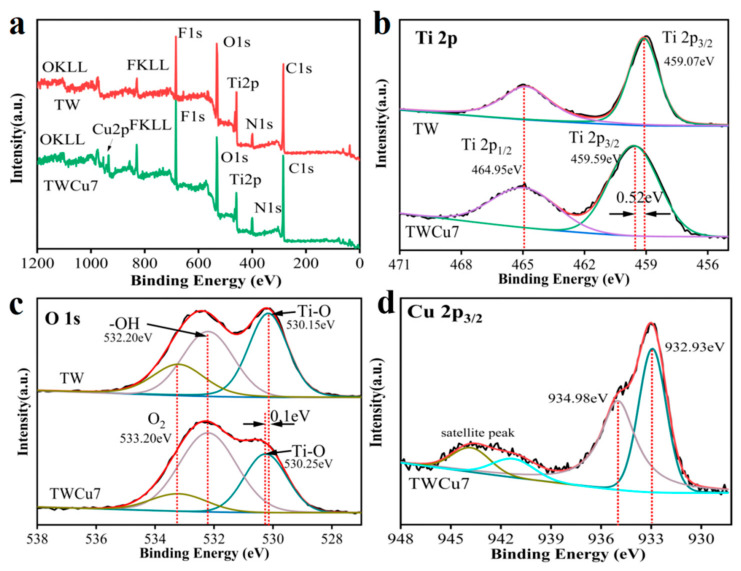
(**a**) XPS spectra of survey scan and XPS high-resolution spectra of (**b**) Ti 2p, (**c**) O 1s, and (**d**) Cu 2p_3/2_.

X-ray photoelectron spectroscopy (XPS) was employed to investigate the surface chemical composition of the sample. As shown in Figure 3a, C, O, N, F, and Ti were detected in TW, while C, O, N, F, Ti, and Cu were detected in TWCu7, indicating that TWCu7 was successfully doped with Cu. The presence of C could be attributed to the wood substrate or other carbon-based impurities, while the N and F could come from the (NH_4_)_2_TiF_6_ precursor. Figure 3b presents the high-resolution XPS spectra of Ti 2p of the TW and TWCu7 samples. The Ti 2p core-level spectrum of TW displayed two peaks at 464.95 eV and 459.07 eV, corresponding to Ti 2p_1/2_ and Ti 2p_3/2_, respectively. The binding energy difference between the two peaks of TW was approximately 5.88 eV, indicating the presence of the Ti^4+^ oxidation state. The Ti 2p_1/2_ and Ti 2p_3/2_ peaks of TWCu7 were observed at 464.95 eV and 459.59 eV, respectively. Nevertheless, the energy at Ti 2p_3/2_ observed in the TW sample was approximately 0.52 eV lower than that of the TWCu7 sample. The change in E_b_ could be attributed to the contact between the Ti^4+^ host ions and the doped Cu ions, indicating the formation of a certain interaction between Cu and TiO_2_, corroborating the literature [35,36]. Figure 3c presents the high-resolution XPS spectra of O 1s in the TW and TWCu7 samples. The peaks at 530.15, 532.20, and 533.20 eV were assigned to Ti–O, the hydroxyl groups on the wood’s surface, and the adsorption of oxygen in water molecules or carbon–oxygen bonds, respectively [37,38,39]. The binding energy of the Ti–O bond in the TWCu7 sample was 0.1 eV higher than that in the TW sample. This change in binding energy indicates that the following electron density of O in the samples had changed after doping with Cu and Ti–O–Cu bonds were formed [40]. Therefore, there was an unusual electron transfer behavior between the Ti^4+^ and Cu ions, which explained the successful doping of Cu into the TiO_2_ nanoparticles. Figure 3d indicated the high-resolution spectrum of Cu 2p_3/2_. The distinctive peak at 932.93 eV was the characteristic peak of Cu 2p_3/2_ for Cu^+^. The peak at 934.98 eV was the characteristic peak of Cu 2p_3/2_ and was assigned to Cu^+^. In addition, the Cu on the TWCu7 surface displayed a high degree of redox reversibility between Cu^2+^ and Cu^+^, which was conducive to the progress of the photocatalytic reaction process [41]. The Cu and the binding energy transition of Ti 2p_3/2_ were observed by XPS, which confirmed that Cu had been doped successfully into the TiO_2_ nanoparticles.

Thermogravimetric analysis (TGA) was performed to illustrate the mass loss rate of each sample. Figure S2 presents the TGA curves of the original wood, TW, and TWCu7 samples. At the end of the final test, the mass loss of the original wood, TW, and TWCu7 was 100%, 98.70%, and 97.52%, respectively. The remaining mass of the TWCu7 sample was higher than that of TW, which was attributed to doped Cu, confirming successful Cu doping.

### 2.2. Optical Properties

**Figure 4:**
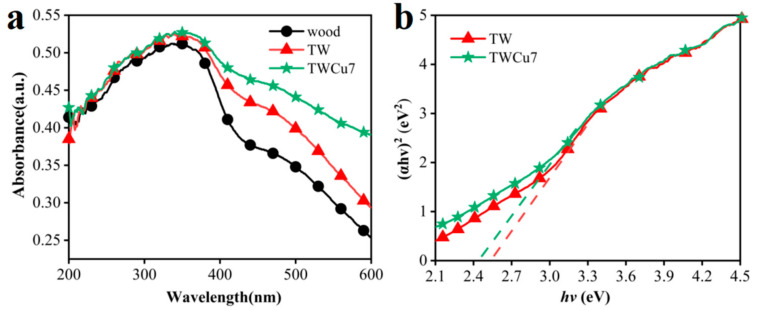
UV-Vis diffuse reflectance (**a**) spectra and (**b**) bandgap energies of wood, TWm and TWCu7 samples.

Herein, UV-Vis diffuse reflectance spectroscopy was performed. The corresponding bandgap energies of the TW and TWCu7 samples were obtained to determine if a sample doped with Cu could expand the response range of light, as shown in Figure 4. These materials showed absorption in the UV and visible regions (Figure 4a). The light absorption of the TW and TWCu7 samples in the range of 200–600 nm was much higher than that of the original wood sample, meaning that the UV-Vis absorption performance of the modified wood was superior to that of the original wood. Compared with TW, the light absorption of the TWCu7 sample underwent some degree of redshift, which broadened the light absorption range. This redshift may be attributed to the doped Cu ions forming a donor level above the initial valence band of TiO_2_, reducing the bandgap of TiO_2_. The bandgap of the sample was calculated using the Kubelka–Munk function, and the results are presented in Figure 4b. Compared with TW, the bandgap of the TWCu7 sample was narrower, confirming that the reason for the redshift mentioned above was correct.

### 2.3. Photocatalytic Activity

**Figure 5:**
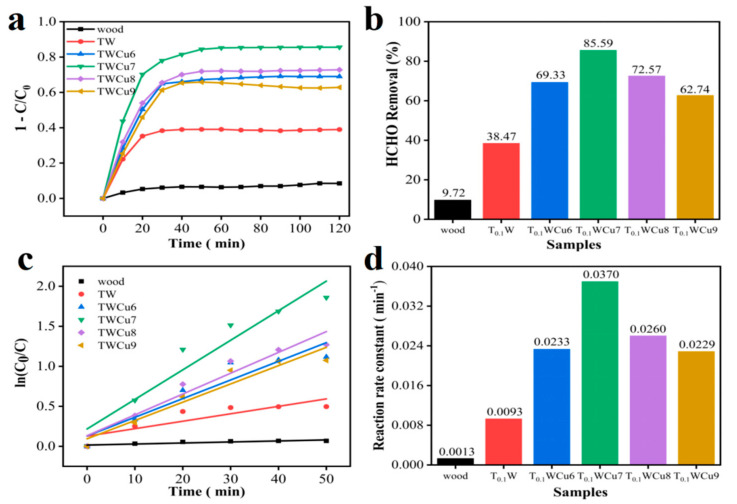
Two-hour photocatalytic reaction degradation efficiency (**a**) process and (**b**) results. (**c**) The kinetic curves and (**d**) the reaction rate constant values.

The photocatalytic activity of the synthesized sample was assessed by degrading the amount of gaseous formaldehyde under ultraviolet-visible light. A series of studies was carried out to investigate the influence of Cu doping on the photocatalytic performance of wood-based composite materials. Figure 5a compares the photocatalytic formaldehyde degradation of the samples with varying Cu concentrations within 2 h. Without any samples, the formaldehyde degradation efficiency was nearly zero within 2 h after turning on the light. The degradation rate of formaldehyde was about 9.72% after 2 h of illumination in the presence of the pure wood sample without a photocatalyst. This was attributed to the adsorption of formaldehyde molecules via the porous structure of wood and the abundant hydroxyl groups on the surface. Meanwhile, the photocatalytic activity of the pure TiO_2_-coated wood samples under UV-Vis light was lower than that of the Cu-doped TiO_2_ wood samples. The formaldehyde degradation efficiency of the pure TiO_2_ wood samples within 2 h was 38%, whereas it was 69.33%, 85.59%, 72.57%, and 62.74% for the TWCu6, TWCu7, TWCu8, and TWCu9 samples, respectively (Figure 5b). As the Cu level increased, the photocatalytic degradation efficiency of the sample first increased and then decreased. Thus, the sample with a Cu/Ti molar ratio of 7% had the highest photocatalytic degradation efficiency (85.59%). Figure 5c shows the fitted models of photocatalytic removal for formaldehyde, which agreed well with the pseudo-first-order kinetics. Figure 5d shows the kinetic constants of Figure 5c. As shown in Figure 5d, the photocatalytic reaction rate constant of TWCu7 (0.0370 min^−1^) under UV-visible light irradiation was also greater than that of the other photocatalysts, being approximately 28.46, 3.98, 1.59, 1.42, and 1.62 times those of wood, TW, TWCu6, TWCu8, and TWCu9, respectively. An appropriate amount of Cu ions could be used as an intermediate for light-generated holes, electron transfer, and preventing hole–electron recombination. Therefore, the doping of Cu can substantially enhance the photocatalytic activity of TiO_2_ under UV-Vis irradiation, which is also the cause of the high degradation efficiency of Cu–TiO_2_/wood composites.

### 2.4. Recyclability of Cu-TiO_2_/Wood Composites

**Figure 6:**
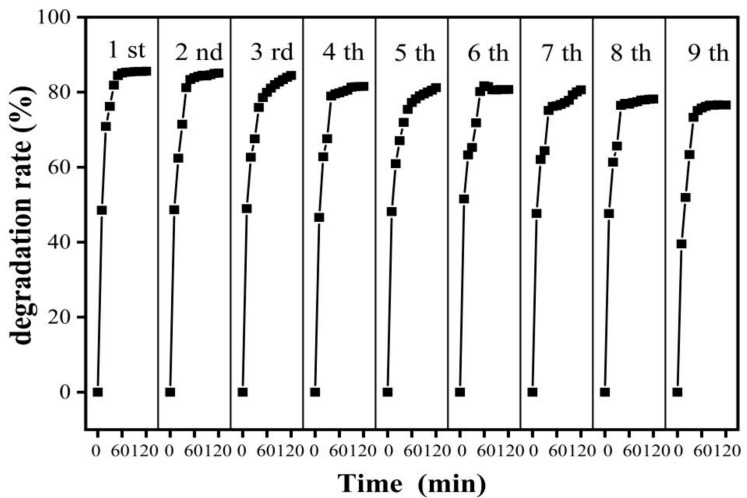
Nine cycles of the photocatalytic behaviors of TWCu7 for formaldehyde under UV-Vis light for 2 h.

In practical applications, the recyclability of TWCu7 is also important. Therefore, the stability and recyclability of the TWCu7 sample were tested. The photocatalytic performance of the TWCu7 sample decreased with the increasing number of times recycled (Figure 6). The photocatalytic efficiency of the TWCu7 sample was more than 80% after 7 cycles and 76.59% after 9 cycles. The photocatalytic efficiency of the sample did not vary significantly, suggesting that the photocatalyst enhanced with Cu ions on a wood substrate had outstanding photochemical stability. Moreover, it was a promising and effective way to remove formaldehyde from indoor air. The enhanced stability of TWCu7 maybe due to the following two reasons: (1) efficient accelerated separation between photoinduced electron–hole pairs, or (2) during the photodegradation process, the porous structure of wood can offer faster gas diffusion for the product, preventing aggregation of the product and photocatalyst deactivation.

### 2.5. Summary of Photocatalytic Mechanism

**Figure 7:**
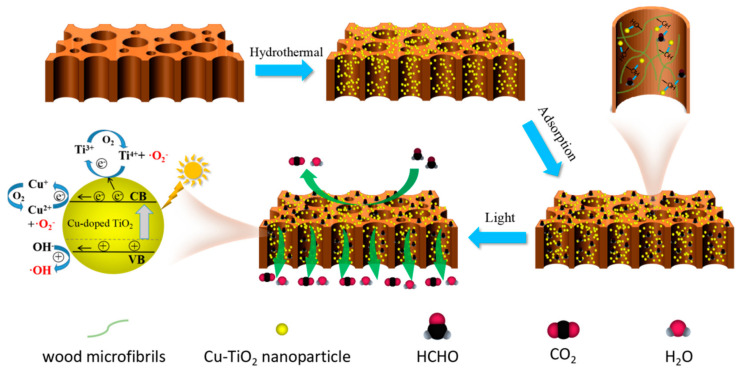
Schematic diagram for Cu-doped TiO_2_ on wood photocatalytic degradation of formaldehyde.

To further depict the process of formaldehyde degradation with Cu-TiO_2_/wood composites more clearly, Figure 7 shows a schematic representation of the process mechanism for the photocatalytic degradation of formaldehyde. First, the wood with abundant hydroxyl groups was used as the reaction substrate material, which was fully combined with Cu ions and Ti ions in the hydrothermal reaction to form a Cu-TiO_2_/wood composite with rich, active sites.

Then, formaldehyde was adsorbed via the numerous hydroxyl groups on wood through hydrogen bonding. As we all know, TiO_2_ nanoparticles have an energy band structure. When exposed to light with an energy greater than or equal to its bandgap energy, the electrons (e^−^) in the valence band (VB) are excited to the conduction band, leaving holes (h^+^) in the conduction band (CB). After Cu doping, a new energy level is formed between CB and VB. The electrons in the VB of TiO_2_ can be excited to transition to the impurity level Cu^2+^/C^+^ and then further transfer to the CB through the secondary excitation of visible light. Therefore, Cu^2+^ might be an electron capture center in the TiO_2_ conduction band, which could be transformed into Cu^+^ by capturing photogenerated electrons. While Cu^+^ was unstable, it reduced O_2_ to a superoxide anion (O_2_^−^) by donating an electron to O_2_ adsorbed on the TiO_2_ surface through the oxidation of Cu^+^ to Cu^2+^. In addition, a portion of the e^−^ was transferred to the Ti^4+^ on the surface to generate Ti^3+^, and the adsorbed O_2_ was then reduced to**·**O_2_^−^. Similarly, h^+^ would convert the hydroxyl groups on wood into hydroxyl radicals (**·**OH). When the molar ratio of doped Cu was lower than its proper molar ratio, Cu doping primarily occurred on the sample surface, which produced the electrons and holes and inhibited the recombination of holes and electrons produced by light. However, when the amount of Cu doping was significantly higher than its appropriate molar ratio, the excess Cu ions were collected mainly below the semiconductor surface, which would also inhibit light-excited electrons and holes. The photocatalytic formaldehyde degradation performance of the prepared sample was lowered. Therefore, proper Cu doping can reduce the photogenerated hole–electron recombination rate and improve the photocatalytic performance. Hence, the transfer of charges was improved, and the recombination of excited electrons and holes was suppressed due to the well-known Schottky barrier effect [42]. Finally, the O_2_^−^ and OH generated via the photocatalyst would break down formaldehyde to CO_2_ and H_2_O under intense oxidation.

Compared with the pure Cu-TiO_2_ powder, the Cu-TiO_2_ nanoparticles anchored on wood exhibited much greater dispersion and a larger active area. In addition, abundant hydroxyl groups on wood can also provide abundant reactive groups for the breakdown of formaldehyde. The porous structure of wood is an excellent channel for moving gas molecules and preventing gas accumulation on the wood, reducing the photocatalytic performance. In this process, it benefited from the synergistic effects of natural wood and the Cu-TiO_2_ photocatalyst.

## 3. Materials and Methods

### 3.1. Materials and Chemicals

All the chemical agents were analytical-grade and used without further purification. All water used was ultrapure water. Poplar wood was purchased from Shijiazhuang, Hebei (China). Urea (CO(NH_2_)_2_), a formaldehyde aqueous solution (HCHO), and copper sulfate pentahydrate (CuSO_4_·5H_2_O) were provided by Sinopharm Chemical Reagent Co., Ltd. Ammonium fluorotitanate ((NH_4_)_2_TiF_6_) was obtained from Shanghai Macklin Biochemical Co., Ltd. The size of the wood sample was 20 mm (L) × 20 mm (R) × 1.5 mm (T), and it was ultrasonically cleaned with ultrapure water for 30 min and then dried in an oven at 45 °C for 24 h.

### 3.2. Preparation of Cu-TiO_2_/Wood Composites

A typical synthetic procedure was performed as follows. The titanium sources were provided by ammonium fluorotitanate. Briefly, 0.1 M (NH_4_)_2_TiF_6_ and 0.02 M CO(NH_2_)_2_ were prepared with water as the solvent. Then, 0.1 M (NH_4_)_2_TiF_6_, 0.02 M CO(NH_2_)_2_, and copper sulfate pentahydrate were combined and stirred magnetically for 10 min. The wood samples and the solution were transferred to a stainless steel autoclave lined with polytetrafluoroethylene and baked at 90 °C for 3 h. After cooling naturally to room temperature, the wood samples were removed and washed repeatedly with ultrapure water to remove the residual solution on the surface. Finally, the material was placed in an oven at 45 °C for 6 h. Products with different copper contents (6%, 7%, 8%, and 9% in terms of the n_Cu_/n_Ti_ molar ratio) were evaluated to determine the influence of copper doping on the phase structure and physical and chemical properties of the sample. In this work, the pure TiO_2_-anchored wood samples are labeled TW, and the Cu-doped TiO_2_ wood samples are represented by TWCuX (X is the percentage of Cu).

### 3.3. Characterization

Scanning electron microscopy (SEM, Zeiss Sigma 300, Germany) and energy dispersive X-ray analysis (EDS) were used to characterize the morphological features and element detection of the samples. The transmission electron microscope (TEM) image of the prepared sample was taken on the FEI TalosF200x (USA) electron microscope at an accelerating voltage of 200 kV. The phase of the samples was determined by X-ray diffraction (XRD, Bruker D8 Advance, Germany) measurement using Cu Ka radiation (λ = 1.5406 Å) at a scanning rate (2θ) of 2°/min in the degree range from 10° to 80°. The X-ray photoelectron spectroscopy (XPS, Thermo Scientific K-Alpha, USA) was scanned with a monochromatic Al-Kα X-ray source at a working voltage of 12 kV, and the surface chemistry of the sample was analyzed. The optical properties of the samples were characterized by ultraviolet-visible diffuse reflectance spectroscopy (UV-vis DRS, Shimadzu 3600 plus, Japan).

### 3.4. Photocatalytic Activity

A schematic diagram of a photocatalytic reaction device is shown in Figure S3. The test was carried out at room temperature in a closed dryer with a volume of approximately 3 L. A 300 w xenon lamp (PLS-SXE300+, Beijing Perfectlight Technology Co. Ltd., Beijing, China) was used for irradiation, and the distance between the sample and the lamp was 15 cm. The photocatalytic performance of the sample was evaluated by monitoring the degradation of formaldehyde under ultraviolet-visible light. Initially, formaldehyde could not be detected in the closed dryer with a formaldehyde detector. In a dark environment, we put the prepared 4 samples and 0.3 μm of the formaldehyde solution into the dryer, using the formaldehyde solution to volatilize formaldehyde gas, and rotated the tiles at the same time for 30 min to reach adsorption equilibrium. Without the sample, the formaldehyde content that reached the adsorption equilibrium was about 11 ppm. After equilibration, the initial concentration of formaldehyde in the airtight dryer was measured with a formaldemeter (PPM-HTV-M, U.K.). Finally, the xenon lamp was turned on to irradiate the sample. The duration of each group of xenon lamp irradiation was 2 h. The formaldehyde degradation effect of the sample was evaluated according to the following formula:

(3) $$D\left(\%\right)=\frac{C_{0}-C}{C_{0}}\times 100\%$$

where *C*_0_ and *C* stand for the initial concentration before illumination and the reaction concentration of formaldehyde after illumination, respectively.

### 3.5. Recyclability of Cu-TiO_2_/Wood Composites

After each cycle of 2 h, we took the sample out and placed it in an oven at 45 °C for 1 h, avoiding light during this period, and performed a new cycle.

## 4. Conclusions

In conclusion, Cu–TiO_2_/wood composites were prepared using a one-step hydrothermal method and applied to the UV-Vis photocatalytic degradation of formaldehyde. The Cu-doped, TiO_2_-modified wood exhibited enhanced photocatalytic activity, mainly due to the synergistic action of wood and Cu–TiO_2_ photocatalysts. Doping with Cu decreased the photogenerated hole–electron recombination rate of TiO_2_, which increased the photoresponse range and improved the photocatalytic performance. The photocatalytic degradation of formaldehyde within 2 h was the highest (85.59%) when the Cu-to-Ti molar ratio was 7%. In addition, the Cu-doped, TiO_2_-modified wood showed outstanding photochemical stability and reusability, with a degradation rate of more than 80% after 7 cycles. This study suggests a new approach for developing UV-Vis light-driven wood-based photocatalysts to reduce indoor formaldehyde emissions from wood furniture.

## Data Availability

Not applicable.

## Supplementary Materials

The following supporting information can be downloaded at: https://www.mdpi.com/article/10.3390/molecules28030972/s1, Table S1: Photocatalytic performances of various modified TiO_2_ based catalysts; Figure S1: EDS spectra of samples; Figure S2: TG of the original wood, TW, and TWCu7 samples; Figure S3: Schematic diagram of photocatalytic reaction device.
