# Supplementary material for: Synthesis of Cu-Doped TiO2 on Wood Substrate with Highly Efficient Photocatalytic Performance and Outstanding Recyclability for Formaldehyde Degradation
Source: Molecules. 2023 Jan 18;28(3):972. doi: 10.3390/molecules28030972 (PMC9921009; doi:10.3390/molecules28030972)
Supplement: Supplementary file 1 [file molecules-28-00972-s001.zip › molecules-2116065-supplementary.pdf]

Support information

Synthesis of Cu-doped TiO_2 on wood substrate with highly efficient photocatalytic performance and outstanding recyclability for formaldehyde degradation

Zhiqiang Lv, Yi Ma, Shanshan Jia, Yan Qing*, Lei Li, Yangyang Chen, Yiqiang Wu*.

College of Materials Science and Engineering, Central South University of Forestry and Technology, Changsha 410004, P. R. China.

* Corresponding authors

Yan Qing

Affiliation: College of Materials Science and Engineering, Central South University of Forestry and Technology, Changsha, Hunan 410004, China

Email address: qingyan0429@163.com

Phone number: +86 731 85623301

Fax: +86 731 85623301

Yiqiang Wu

Affiliation: College of Materials Science and Engineering, Central South

University of Forestry and Technology, Changsha, Hunan 410004, China

Email address: wuyq0506@126.com

Phone number: +86 731 85623989

Fax: +86 731 85623989

Table S1 Photocatalytic performances of various modified TiO_2 based catalysts.

Samples	Type	Modified elements	Light source	Pollutant	Pollutant concentration	Degradation rate	Reference
TiO_2	Composites	ONLH (the oxygen or nitrogen linked heptazine-base polyme)	Natural sunlight	medicine	10 mL/g	$(14.4 \pm 0.5) \times 10^{-2}$ min^{-1}	[11]
TiO_2	Composites	Natural silicate	UV	HCHO	30 ppm	0.7 $\mu mol \cdot s^{-1} \cdot g^{-1}$	[12]
TiO_2	Composites	kaolinit	UV	Ciprofloxacin	10 mg/L	0.43 min^{-1}	[13]
TiO_2	Doping	Fe-I	Visible light	Benzene	100 mg/m^3	59.38%	[14]
TiO_2	Doping	La/Mn	UV light	MB	20 mg/L	96.44%	[16]
TiO_2	Composites	NH_2-MIL-125	UV	HCHO	10 ppm	90%	[17]

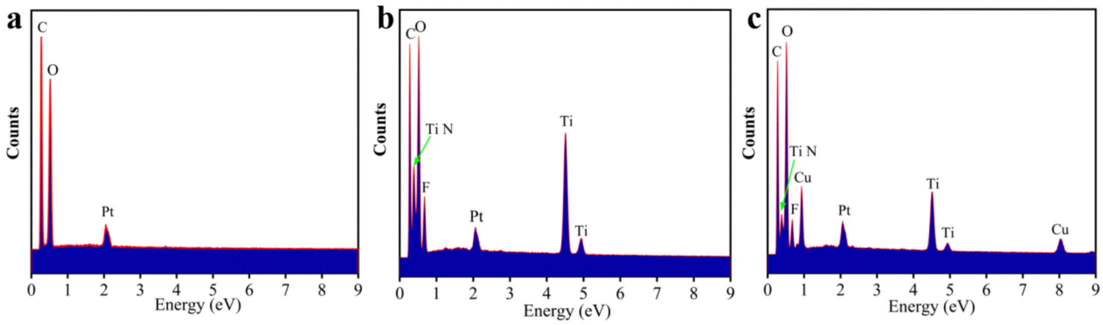

Figure S1. EDS spectra of samples: (a) wood; (b) TW; (c) TWCu7.

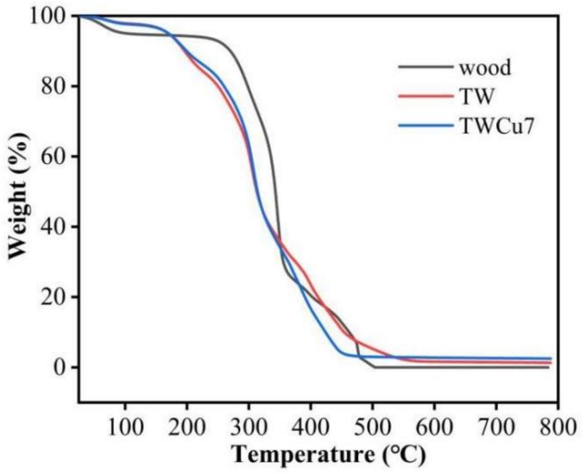

Figure S2. TG of the original wood, TW, and TWCu7 samples.

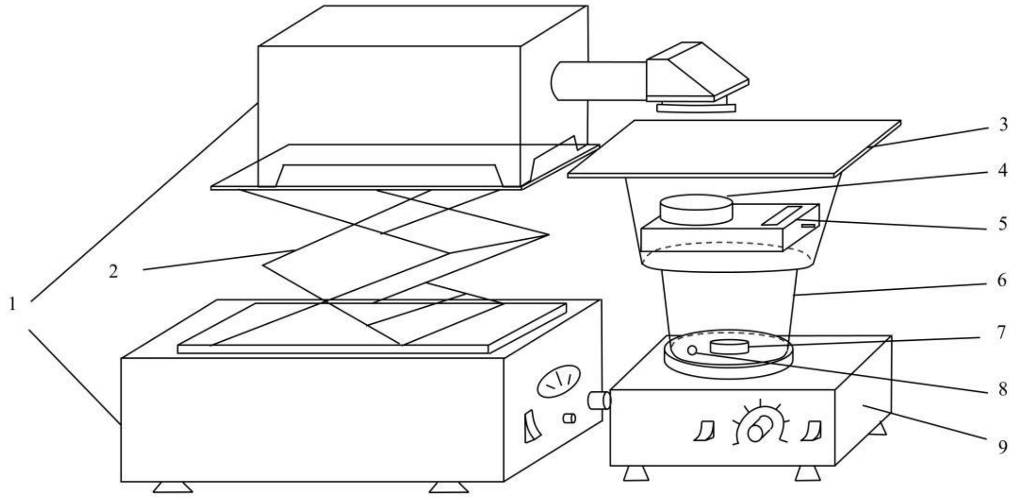

Figure S3. Schematic diagram of photocatalytic reaction device. 1. Xenon lamp house; 2. Lift table; 3. PMMA; 4. Sample; 5. PPM HTV m; 6. Drying apparatus; 7. Magnetic rotor; 8. Formaldehyde liquid; 9. Magnetic stirrer.
